# Ex Vivo Apoptosis in CD8+ Lymphocytes Predicts Rectal Cancer Patient Outcome

**DOI:** 10.1155/2016/5076542

**Published:** 2016-06-01

**Authors:** Sebastian Winkler, Philipp Hoppe, Marlen Haderlein, Markus Hecht, Rainer Fietkau, Luitpold V. Distel

**Affiliations:** Department of Radiation Oncology, University Hospital Erlangen and Friedrich-Alexander-University Erlangen-Nürnberg, 91054 Erlangen, Germany

## Abstract

*Background*. Apoptotic rates in peripheral blood lymphocytes can predict radiation induced normal tissue toxicity. We studied whether apoptosis in lymphocytes has a prognostic value for therapy outcome.* Methods*. Lymphocytes of 87 rectal cancer patients were ex vivo irradiated with 2 Gy, 8 Gy, or a combination of 2 Gy ionizing radiation and Oxaliplatin. Cells were stained with Annexin V and 7-Aminoactinomycin D and apoptotic and necrotic rates were analyzed by multicolor flow cytometry.* Results*. After treatment, apoptotic and necrotic rates in CD8+ cells are consistently higher than in CD4+ cells, with lower corresponding necrotic rates. Apoptotic and necrotic rates of CD4+ cells and CD8+ cells correlated well within the 2 Gy, 8 Gy, and 2 Gy and Oxaliplatin arrangements (*p* ≤ 0.009). High apoptotic CD8+ rates after 2 Gy, 8 Gy, and 2 Gy + Oxaliplatin treatment were prognostically favorable for metastasis-free survival (*p* = 0.009, *p* = 0.038, and *p* = 0.009) and disease-free survival (*p* = 0.013, *p* = 0.098, and *p* = 0.013).* Conclusions*. Ex vivo CD8+ apoptotic rates are able to predict the patient outcome in regard to metastasis-free or disease-free survival. Patients with higher CD8+ apoptotic rates in the peripheral blood have a more favorable prognosis. In addition to the prediction of late-toxicity by utilization of CD4+ apoptotic rates, the therapy outcome can be predicted by CD8+ apoptotic rates.

## 1. Background

A radiochemotherapeutic treatment induces cell death in cancer cells and normal tissue mainly by apoptosis and necrosis [1]. There have been numerous attempts to correlate apoptotic rates in the cancer tissue-sections to the prognosis of cancer patients. However, they had varying success in finding a correlation between high apoptotic rates and a favorable outcome [2–6]. In several studies, this association failed to be demonstrated. Another approach is to predict individual radiosensitivity by apoptotic rates. Since 1996, Crompton and  Ozsahin propagated the prediction of individual radiosensitivity and by implication normal tissue toxicity based on a CD4+ and CD8+ lymphocyte assay [7, 8]. Low rates of apoptotic cells after ex vivo irradiation were found to be able to predict individual radiosensitivity. In a prospective study with 399 cancer patients, they were able to show that apoptosis is capable of predicting ionizing radiation induced late sequelae [9]. Recently, in a prospective prostate cancer study including 214 patients, it had been demonstrated that low CD4+ apoptotic rates are associated to late genitourinary toxicity. In a further study, apoptotic rates in 58 cervix cancer patients were analyzed [10]. These studies by Ordoñez et al. and Foro et al. have added an entirely new aspect. They were the first to show an association between high rates of CD8+ apoptosis and an improved overall survival [10, 11]. Our approach was to analyze whether CD4+ or CD8+ apoptosis and necrosis have a prognostic impact on tumor progression and metastasis in a prospective study of 87 homogenous treated rectal cancer patients.

## 2. Methods

### 2.1. Study Participants

The study included 87 patients which were treated with neoadjuvant radiochemotherapy and surgery. The patients' ages were between 23 and 85 with a mean age of 63.6 years.  Table 1 shows an overview of the distribution of radiotherapy, chemotherapy, and surgery for all individuals. This study was approved by the ethics review committee of the Friedrich-Alexander-Universität Erlangen-Nürnberg (Number 2725) and all patients and healthy individuals gave their written informed consent. Blood samples were collected shortly before the first irradiation treatment.

### 2.2. Lymphocyte Isolation

Prior to the first radiotherapy treatment, 3 mL of heparinized whole peripheral blood was collected from each of the patients. Lymphocytes were freshly isolated through Ficoll-Paque density gradient (Biochrom, Berlin, Germany) centrifugation for 15 minutes at 1200 G and 24°C. Subsequently, the mononuclear cell layer was harvested and these cells were washed by resuspension with RPMI 1640 medium (Sigma-Aldrich, Taufkirchen, Germany) and centrifugation for 10 minutes at 300 g and 24°C. This last step was repeated once. The washed cells were added to a nutrient medium consisting of 7.2 mL RPMI 1640 medium, 1.8 mL fetal bovine serum (Biochrom), 100 *μ*L Penicillin (10.000 U/mL), and Streptomycin (10.000 *μ*g/mL) (Gibco, Life Technologies, Darmstadt, Germany). The cell suspension was divided into ten 1.4 mL U-bottom push cap tubes (Micronic, Wernberg-Köblitz, Germany) with 400 *μ*L each.

### 2.3. Irradiation and Treatment with Chemotherapeutics

Three tubes were left untreated, three were ex vivo irradiated with 2 Gy, and two were irradiated with 8 Gy ionizing radiation. Cells were irradiated at a dose rate of 2 Gy per minute with a 120 kV X-ray machine (ISOVOLT Titan; GE, Ahrensburg, Germany). Furthermore, cells in two U-bottom push cap tubes were incubated for 0.5 h with 10 *μ*g/mL Oxaliplatin (pharmacy of the University Hospial, Erlangen, Germany) and afterwards irradiated with 2 Gy ionizing radiation. Cells were incubated for further 48 h with Oxaliplatin in the 1.4 mL U-bottom tubes at 37°C before flow cytometry analysis.

### 2.4. Flow Cytometry

**Figure 1:**
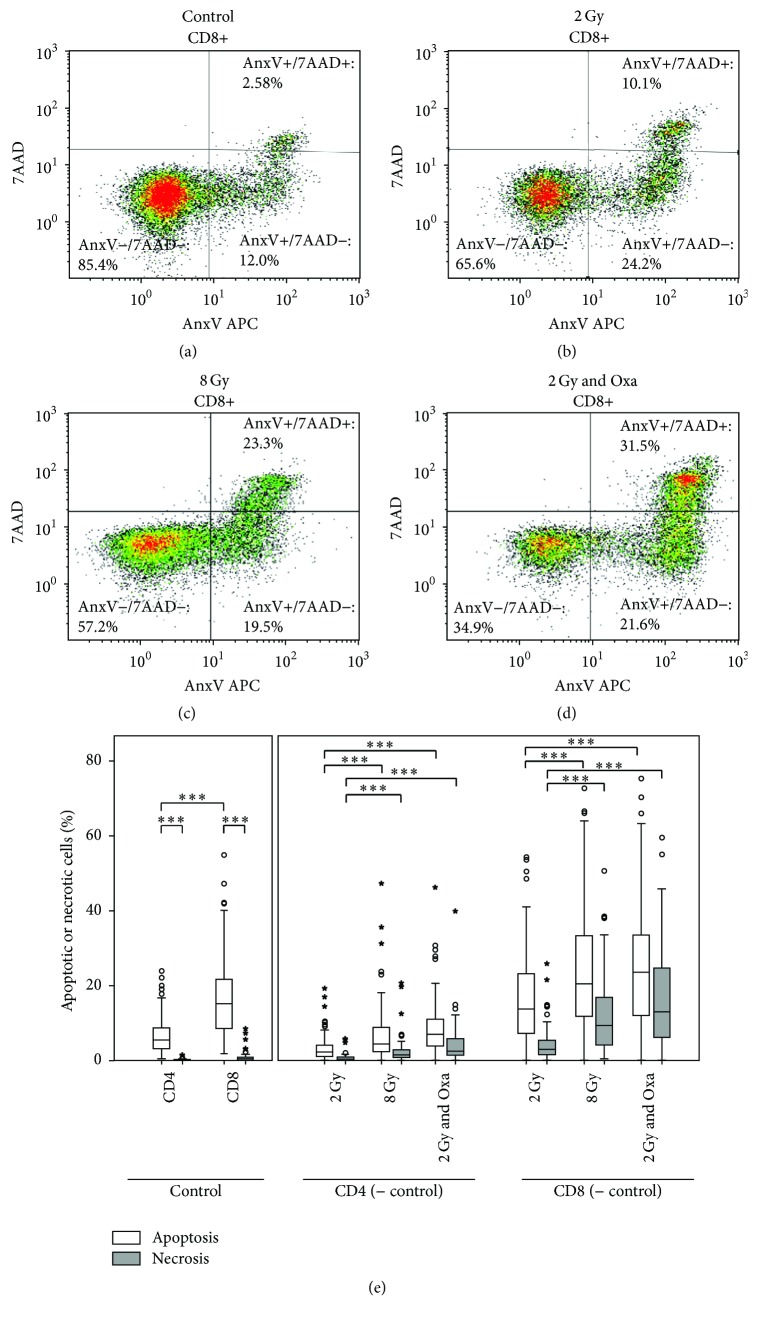
Evaluation of apoptotic and necrotic rates of CD8+ cells with the cell analysis software Kaluza, showing an example of (a) untreated samples, (b) samples treated with 2 Gy ionizing radiation, (c) samples treated with 8 Gy ionizing radiation, and (d) samples treated with 2 Gy ionizing radiation combined with 10 *μ*g/mL Oxaliplatin. (e) Apoptotic (white bars) and necrotic (grey bars) rates in percentages by treatment and lymphocyte subgroups CD4+ and CD8+ of 87 rectal cancer patients. Control values represent the apoptotic or necrotic background and were subtracted from the values of the treated samples. Significantly different values were marked by three asterisks (*p* < 0.001).

After incubation of lymphocytes for 48 h at 37°C and 5% CO_2_, the samples were centrifuged for 10 minutes at 300 G and 4°C, and then put on ice and decanted. Each pellet was resuspended in 200 *μ*L lactated ringer's solution. A mix of monoclonal antibodies, Annexin V-APC (BD Biosciences, Heidelberg, Germany) and 7-Aminoactinomycin D (7AAD) (BD Biosciences, Heidelberg, Germany), was added, consisting of the following quantities: 20 *μ*L of CD4-FITC, 20 of *μ*L CD8-PE, 5 *μ*L of Annexin V-APC, and 5 *μ*L of 7-Aminoactinomycin. After 30 minutes on ice, another 200 *μ*L of lactated ringer's solution (Braun, Melsungen, Germany) was added to each sample and lymphocytes were analyzed using a Gallios flow cytometer (Beckmann Coulter, Krefeld, Germany). In order to identify lymphocytes, forward scatter and sideward scatter were used. Lymphocyte subtypes were identified according to their CD4+ and CD8+ surface antigens. Apoptotic and necrotic rates were measured by Annexin V and 7AAD staining (Figures 1(a)–1(d)).

### 2.5. Statistical Analysis

The raw data of flow cytometry were analyzed by the analysis software Kaluza 1.1 (Beckman Coulter, Krefeld, Germany). Here, the subgroups of lymphocytes as well as the apoptotic and necrotic rates of each of these subgroups were determined. Then, the data was transferred to Excel (Microsoft, Redmond, WA, USA), tabulated and transferred to SPSS version 22 (IBM, Ehningen, Germany), and statistically analyzed. The rates of apoptosis and necrosis used in the statistical analysis are the apoptotic and necrotic rates of the treated lymphocytes and subtracted basic apoptotic or necrotic rates of the untreated lymphocytes. The analysis of metastasis-free survival (MFS) and disease-free survival (DFS) was calculated according to the Kaplan-Meier method. Differences in survival were assessed with the log-rank test. The level of significance was defined as *p* ≤ 0.05.

## 3. Results

The apoptotic and necrotic rates were determined for ex vivo irradiated CD4+ and CD8+ peripheral blood lymphocytes (Figures 1(a)–1(d)) in 87 rectal cancer patients. All patients were intended to receive a neoadjuvant radiochemotherapy followed by total mesorectal resection (Table 1). Basic apoptotic and necrotic rates from untreated cells were subtracted from apoptotic and necrotic rates of ex vivo treated lymphocytes (Figure 1(e)). Lymphocytes were ex vivo treated with 2 Gy and 8 Gy ionizing radiation or a combination of 2 Gy and 10 *μ*g/mL Oxaliplatin. CD8+ cells sustained higher rates of apoptosis and necrosis than CD4+ cells (*p* < 0.001). Apoptosis invariably exceeds necrosis (*p* < 0.003). The rates of apoptosis and necrosis increase for both CD4+ and CD8+ cells when treated with a higher dose of ionizing radiation and considerably when treated with the combination of radiation and Oxaliplatin (Figure 1(e)).

**Figure 2:**
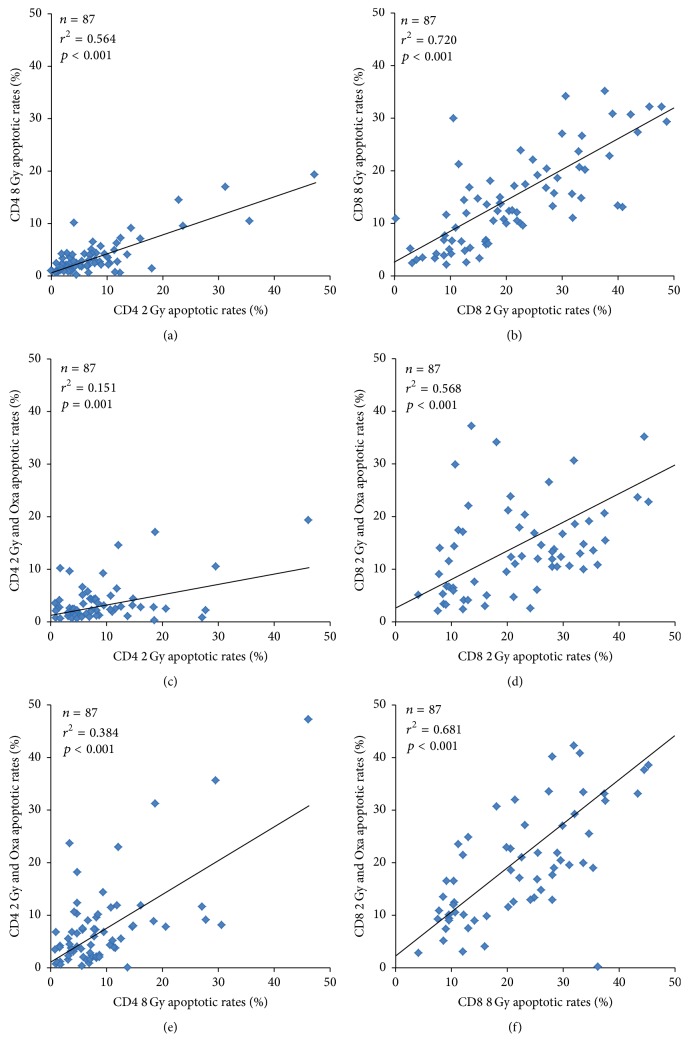
Correlation of 87 rectal cancer patients CD4+ (a, c, and e) or CD8+ (b, d, and f) cells' apoptotic rates regarding the 2 Gy (a and b), 8 Gy (c and d), and combination of 2 Gy and 10 *μ*g/mL Oxaliplatin (e and f) treatment. The diagrams include the coefficients of determination (*r* ^2^), *p* value, and the trend line.

Apoptotic rates of CD4+ cells and CD8+ cells correlated well within the 2 Gy, 8 Gy, and 2 Gy + Oxaliplatin arrangements (Figure 2) (*p* ≤ 0.001), and the same applies for necrotic rates (*p* ≤ 0.009) (Table 2). The correlation between apoptosis and necrosis was much weaker; only in CD4+ cells at 2 Gy (*p* < 0.001) or 8 Gy (*p* = 0.02) was a strong correlation observed. In CD8+ cells, apoptosis only correlated with necrosis after treatment with 2 Gy and Oxaliplatin (*p* = 0.001) (Table 2).

**Figure 3:**
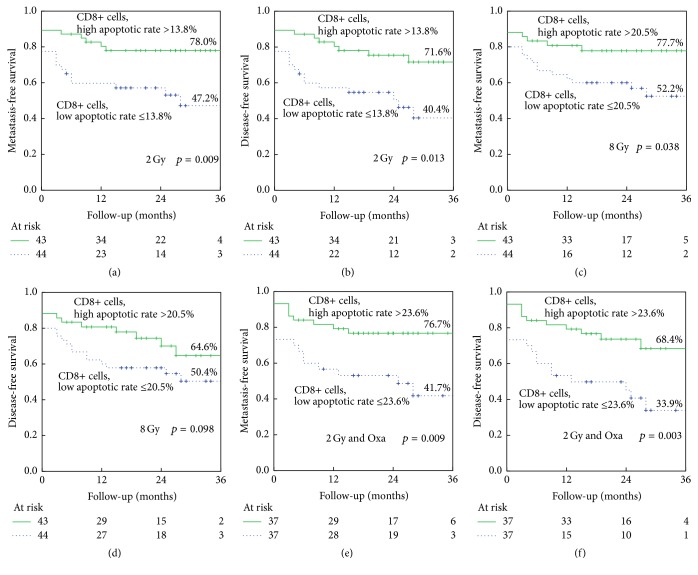
Kaplan-Meier analysis of CD8+ apoptotic rates after 2 Gy (a) metastasis-free survival, (b) disease-free survival, 8 Gy, (c) metastasis-free survival, (d) disease-free survival and 2 Gy and 10 *μ*g/mL Oxaliplatin, (e) metastasis-free survival, and (f) disease-free survival. *p* values are calculated by the log-rank test. Numbers of patients at risk at the times of 0, 12, 24, and 36 months of follow-up and percentage of remaining patients with event-free survival are indicated.

Kaplan-Meier plots were used to compare the patient's prognosis in regard to metastasis-free survival, disease-free survival, overall survival, and tumor-specific survival. Patients were divided into two groups, one including those with apoptotic or necrotic rate above median values and one including those with apoptotic or necrotic rate below median values. High CD8+ apoptotic rates were prognostically favorable for metastasis-free survival (Figures 3(a), 3(c), and 3(e)) and disease-free survival (Figures 3(b), 3(d), and 3(f)). This is true for all three types of ex vivo treatment. After 2 Gy ex vivo irradiation (MFS, *p* = 0.009 and DFS, *p* = 0.013) (Figures 3(a) and 3(b)) and the combination of 2 Gy and Oxaliplatin (MFS, *p* = 0.009, DFS, *p* = 0.013) (Figures 3(c) and 3(d)) the most significant differences were observed. Eight Gy ex vivo irradiation gave less clear differences (MFS, *p* = 0.038 and DFS, *p* = 0.098) (Figures 3(e) and 3(f)).

No clear differences were found for overall and tumor-specific survival, while the rate of CD8+ apoptotic cells after the combined treatment of 2 Gy and Oxaliplatin still shows a tendency (*p* = 0.089) to increase tumor-specific survival (data not shown). Neither the CD4+ cell apoptotic rates nor the CD4+ or CD8+ cell necrotic rates were able to predict the patient outcome of any category.

## 4. Discussion

As far as we are aware of, this is the first time a study points out that the rate of CD8+ apoptotic peripheral blood lymphocytes can predict the metastasis-free or disease-free survival of patients with rectal cancer. Recently, two studies reported very similar results [10, 11]. Ordoñez et al. examined a homogenous radiochemotherapy or radiotherapy treated cohort with 58 stage I and II cervical cancer patients. Apoptotic rates were measured by Annexin V and Propidium Iodide flow cytometry. Patients with higher CD8+ cells apoptotic rates had an improved local disease-free survival, regional disease-free survival, metastasis-free survival, and disease-free survival [10]. Foro et al. studied a cohort of 214 radiotherapy treated prostate cancer patients. A high rate of CD8+ apoptosis was prognostically favorable for overall survival [11]. The largest study of 399 cancer patients on CD4+ and CD8+ apoptosis could predict late-toxicity yet failed to predict therapy outcome. Only in a subgroup of breast cancer and head and neck cancer patients was CD8+ apoptosis prognostically beneficial [9]. This indicates that the predictive value of CD8+ apoptotic rates depends on homogenous cohorts. In our study we could not show a prognostic relevance to overall survival. The reason may be the relatively short follow-up time. After a reasonable follow-up period it might be possible to determine a prognostic value for overall survival. However, especially in rectal cancer, where about a third of patients suffer from distant metastases, a test with the capability to predict an increased risk of metastatic spread could be extremely useful.

Several studies demonstrated that CD4+ apoptotic rates in peripheral blood can predict normal tissue toxicity [7–9, 12, 13]. At present, in cervix cancer [10], prostate cancer [11], rectal cancer (this study), head and neck cancer, and breast cancer [9], there is a strong association between high CD8+ apoptotic rates and a favorable prognosis.

Furthermore, we examined the necrosis rate as this has not been done in previous studies. No prediction of patient outcome was possible for 2 Gy, 8 Gy, and the combination of 2 Gy and Oxaliplatin. The same is true for CD4+ lymphocytes. Similarly, Foro et al. did not find a relationship between CD4+ apoptotic rates and patient survival. They showed “that in vitro radiation-induced apoptosis of CD4+ T lymphocytes assessed before radiation therapy was associated with the probability of developing chronic genitourinary toxicity” [11]. As we did not so far collect the data regarding late-toxicity, we cannot confirm these results.

CD8+ lymphocytes are more susceptible to ionizing radiation as well as to the combination of IR with Oxaliplatin (Figure 1(e)). CD4+ apoptotic rates do not have the ability to predict metastasis or relapse, neither have the necrotic rates. It is understandable that necrosis does not have the capability to be predictive, because necrosis is not actively induced by the cells and should therefore not be genetically controlled. However, it is astonishing that CD4+ cells apoptotic rates fail to be predictive, because in normal tissue toxicity prediction CD4+ cells are superior to CD8+ cells [2, 9, 10]. Overall, it is astonishing how CD8+ lymphocytes are able to predict tumor cell survival, resulting in regrowth and leading to recurrence and metastasis. Cancer cells have changed genetically by a high number of mutations most probably also including mutations in the apoptosis pathways. Therefore, it is difficult to imagine that the CD8+ apoptotic rates are directly related to the cancer cell apoptosis and tumor response.

Ordoñez et al. speculated that TGF-*β*1 as an important player in the regulation of apoptosis could cause low levels of radiation induced apoptosis and subsequently would increase the risk of radiation induced toxicity. This would lead secondarily to a decrease in survival of cervical cancer patients, possibly through modulation of the cytokines associated with cancer progression and metastasis [10]. We think along the same lines that the IR induced CD8+ apoptotic rates in the tumor infiltrating lymphocytes are in anyway related to therapy response. It seems probable that this might be caused by the tumor micro milieu and its related cytokines. Secondarily, the cytokines have a systemic and generalized influence on cells in the blood and will influence the CD8+ apoptosis there. Therefore, the assay predicts the CD8+ apoptotic rates in the tumor infiltrating lymphocytes, which are related to tumor response. A second option mentioned by Ordoñez et al. is tumor hypoxia and its association with a reduced therapy response and the induction of several cytokines [10], especially since rectal cancer contains hypoxic areas similar to cervical cancer. Given the very limited data available, the link between circulating CD8+ cells and tumor response is still entirely ambiguous and further work is necessary to study this effect. Nevertheless, this work and the three recently published studies clearly point towards a link between tumor response and CD8+ apoptosis [2, 9, 10].

The CD4+ lymphocytes also show a wider range of apoptotic and necrotic rates and this might be a reason why we could not show a significant difference concerning the prediction of the patients' outcome when using the rates of apoptosis and necrosis of CD4+ cells. The necrotic rates are consistently lower than the apoptotic rates of each treatment group. It may indicate that the dosing of these treatments is in the correct range and the lymphocytes are not heavily damaged so that they are able to induce programmed cell death.

When comparing apoptotic and necrotic rates of CD4+ and CD8+ cells regarding each of the three treatment options (Table 2), no, or at best weak, correlations could be found. As expected, lymphocytes showing higher apoptotic rates after either radiation or chemotherapy were more susceptible to the respective other treatments. More remarkably, there are strong correlations for CD8+ lymphocytes in comparison to relatively weak correlations measured in CD4+ lymphocytes, especially between radiation and radiation combined with Oxaliplatin (Figure 2). This is probably due to interindividual differences explained by, for example, different genetic disposition [14, 15].

The Annexin V– 7AAD cytometry test used for this study is a simple and fairly cheap blood assay that yields results within 48 hours. It can predict patients' outcome and may also be used to predict late-toxicity [9].

## 5. Conclusions

In conclusion, ex vivo CD8+ apoptotic rates are able to predict the patient outcome in regard to metastasis-free or disease-free survival. Patients with higher CD8+ apoptotic rates in the peripheral blood have a more favorable prognosis. In addition to the prediction of late-toxicity by use of CD4+ and CD8+ apoptotic rates, the therapeutic outcome can be predicted by CD8+ apoptotic rates.

## Figures and Tables

**Table 1:** Patients treatment by chemotherapy, surgery and radiotherapy.

	Number of patients
5FU + Oxaliplatin/5FU/5FU + Oxaliplatin reduced dose/5FU reduced dose	67/12/5/3
Surgery/inoperable/complete remission/rejected surgery	76/8/2/1
Radiotherapeutic dose (Gy): 50.4/45.0/55.8/10.8	75/6/5/1

**Table 2:** Correlation of necrotic rates of the different treatments for CD4+ and CD8+ lymphocytes separately and correlation of apoptosis and necrosis rate of CD4+ and CD8+ cells regarding the three different treatments (*p* values < 0.05 are highlighted in bold).

	CD4+ cells			CD8+ cells		
Correlation of necrotic rates	2 Gy/8 Gy	2 Gy/2 Gy & Oxa	8 Gy/2 Gy & Oxa	2 Gy/8 Gy	2 Gy/2 Gy & Oxa	8 Gy/2 Gy & Oxa

*p* value	**r** ^2^ = 0.810	**r** ^2^ = 0.149	**r** ^2^ = 0.092	**r** ^2^ = 0.606	**r** ^2^ = 0.390	**r** ^2^ = 0.681
	**<0.001**	**0.004**	**0.003**	**<0.001**	**<0.001**	**<0.001**

Apoptosis correlated to necrosis	2 Gy	8 Gy	2 Gy & Oxa	2 Gy	8 Gy	2 Gy & Oxa

*p* value	**r** ^2^ = 0.157	**r** ^2^ = 0.062	*r* ^2^ = 0.026	*r* ^2^ = 0.007	*r* ^2^ = 0.012	**r** ^2^ = 0.134
	**<0.001**	**0.02**	0.167	0.432	0.298	**0.001**
